# Systematic Review and Meta-Analysis of the Diagnostic Accuracy of Mobile-Linked Point-of-Care Diagnostics in Sub-Saharan Africa

**DOI:** 10.3390/diagnostics11061081

**Published:** 2021-06-12

**Authors:** Ernest Osei, Sphamandla Josias Nkambule, Portia Nelisiwe Vezi, Tivani P. Mashamba-Thompson

**Affiliations:** 1 Discipline of Public Health Medicine, School of Nursing and Public Health, University of KwaZulu-Natal, Durban 4001, South Africa; 210501689@stu.ukzn.ac.za (S.J.N.); mgabadeli999@gmail.com (P.N.V.); Mashamba-Thompson@ukzn.ac.za (T.P.M.-T.); 2 Faculty of Health Sciences, Prinshof Campus, University of Pretoria, Pretoria 0084, South Africa

**Keywords:** mHealth devices, diagnosis, accuracy, sensitivity, specificity, sub-Saharan Africa

## Abstract

Mobile health devices are emerging applications that could help deliver point-of-care (POC) diagnosis, particularly in settings with limited laboratory infrastructure, such as Sub-Saharan Africa (SSA). The advent of Severe acute respiratory syndrome coronavirus 2 has resulted in an increased deployment and use of mHealth-linked POC diagnostics in SSA. We performed a systematic review and meta-analysis to evaluate the accuracy of mobile-linked point-of-care diagnostics in SSA. Our systematic review and meta-analysis were guided by the Preferred Reporting Items requirements for Systematic Reviews and Meta-Analysis. We exhaustively searched PubMed, Science Direct, Google Scholar, MEDLINE, and CINAHL with full text via EBSCOhost databases, from mHealth inception to March 2021. The statistical analyses were conducted using OpenMeta-Analyst software. All 11 included studies were considered for the meta-analysis. The included studies focused on malaria infections, *Schistosoma haematobium*, *Schistosoma mansoni*, soil-transmitted helminths, and *Trichuris trichiura*. The pooled summary of sensitivity and specificity estimates were moderate compared to those of the reference representing the gold standard. The overall pooled estimates of sensitivity, specificity, positive likelihood ratio, negative likelihood ratio, and diagnostic odds ratio of mobile-linked POC diagnostic devices were as follows: 0.499 (95% CI: 0.458–0.541), 0.535 (95% CI: 0.401–0.663), 0.952 (95% CI: 0.60–1.324), 1.381 (95% CI: 0.391–4.879), and 0.944 (95% CI: 0.579–1.538), respectively. Evidence shows that the diagnostic accuracy of mobile-linked POC diagnostics in detecting infections in SSA is presently moderate. Future research is recommended to evaluate mHealth devices’ diagnostic potential using devices with excellent sensitivities and specificities for diagnosing diseases in this setting.

## 1. Introduction

Currently, Sub-Saharan Africa (SSA) bears the highest disease burden worldwide [1]. The high rate of infectious diseases, high recurrence of epidemics, increasing growth of chronic diseases, weak healthcare systems, insufficient funds to support healthcare, limited skilled health professionals, and poor healthcare infrastructure pose a significant challenge in improving healthcare provision in SSA [2,3,4]. Most patients have limited or no access to healthcare clinics and even essential healthcare services [2]. With these challenges, digital health such as mobile health (mHealth) applications have demonstrated their potentials in screening communicable and non-communicable diseases at point-of-care diagnostics globally, including SSA [5,6,7,8]. mHealth technology is considered one of the emerging diagnostic tools or recognized as an enabling technology for disease diagnosis [1,9,10]. In this study, we define mHealth as the use of mobile health devices such as smartphones, tablets, and others as diagnostic tools to diagnose existing disease conditions in patients [11].

The current global outbreak of the novel Severe acute respiratory syndrome coronavirus 2 (SARS-CoV-2) infections has overstretched many healthcare systems, and its implications are still unfolding. With the considerably increasing number of cases and limited available resources, there is a growing need for deployment of scalable solutions such as digital health technologies, including mHealth applications, to monitor and manage the pandemic [5,9]. A recent study in the USA showed that mHealth applications were used to screen healthcare workers for SARS-CoV-2 symptoms to control the spread of the infection [9]. Other studies conducted in the USA, Canada, and Taiwan have also demonstrated the use mHealth for preliminary screening and early detection of possible SARS-CoV-2-infected persons and accelerating linkage to care [10,12,13]. 

We defined disease diagnosis as the process of identifying a health condition, disorder, or problem by a systematic analysis of a patient’s background or history, examining the symptoms, evaluating the test results, and investigating the probable causes [14]. The diagnosis of disease conditions can be performed accurately or inaccurately by health professionals, patients, and other recognized groups. In this study, diagnostic accuracy can generally be defined as the actual results that contain both true positives (sensitivity) and true negatives (specificity) of a disease condition in a population [15]. Diagnostic accuracy can further be described as a test’s ability to discriminate between the target disease condition and health [16]. 

In low- and middle-income countries (LMICs), several mobile health techniques are being utilized to support healthcare delivery. Studies in SSA revealed that mobile health techniques such as short message service (SMS), voice/phone calls, and mobile apps are predominantly employed to support healthcare delivery [3,11,17]. For instance, recently, mobile phone devices are used to capture images that are processed immediately and analyzed using smart algorithms for disease diagnosis [6,7]. In Botswana, mobile phones are used for diagnostics accuracy of photographs of plain film test X-rays digitally [7]. In SSA, healthcare professionals employed the SMS technique for educating and creating awareness on treatment methods, management of diseases, and availability of health services [8]. Similarly, SMS and voice calls are used to remotely monitor chronic conditions, communicate, and train healthcare professionals, track pandemic and epidemic outbreaks, and data collection [8,11]. Additionally, in SSA and other settings, mobile health techniques such as mobile apps allow the community healthcare workers to enter patients’ symptoms into the app, diagnose illness, and give treatment recommendations [2,4,8]. Furthermore, research has demonstrated that mHealth applications like mobile apps are primarily used for collecting clinical data of patients and healthcare systems to assist in formulating health policies [8,18]. Studies have demonstrated that the short message service technique is the most used mHealth application to support healthcare delivery in SSA [19,20,21]. The evidence available shows that most of these mHealth techniques are based on optical detection methods [6,7]. 

Our scoping review aimed at mapping evidence on mHealth applications to diagnose diseases and support treatment procedures by healthcare workers in SSA [22]. The results showed that mHealth applications are available and are being used to support healthcare services by health professionals. The results demonstrated that mHealth applications are being used for diagnosing certain disease conditions in SSA. The results further indicated that mHealth applications are being utilized to manage HIV, TB, cancer, and hypertension cases in SSA [22]. In recent times, mobile health devices have been employed to provide accurate and rapid diagnosis of diseases at POC diagnostics, which is critical to provide effective and life-saving treatments [23,24,25,26]. Other studies have also demonstrated that access to a simple mHealth device at POC diagnostics can potentially transform individuals’ health behavior and improve people’s preventive interventions in hard-to-reach communities [27,28]. Similar studies revealed that mHealth devices had been used in resource-poor settings at POC diagnostics to detect recent infectious Ebola, Severe Acute Respiratory Syndrome (SARS), and Zika viruses to help in the early treatment of such cases [29,30,31,32]. Although the advent of mobile-linked diagnostics at point-of-care in resource-limited settings helps improve access to healthcare and reduce healthcare inequalities [23,24], there is limited evidence on their diagnostic accuracy. Therefore, we performed this systematic review and meta-analysis to evaluate mobile-linked POC diagnostics’ accuracy in SSA. 

## 2. Materials and Methods

The review followed the Preferred Reporting Items requirements for Systematic Reviews and Meta-Analysis (PRISMA) [33]. The Population, Intervention, Comparison, and Outcome (PICO) framework for determining the primary research question eligibility (Table 1) was followed. 

The primary research question was: What is the evidence on the diagnostic accuracy of mobile-linked POC diagnostics in Sub-Saharan Africa? 

### 2.1. Search Strategy 

An electronic search was carried out to identify all relevant published descriptive quantitative studies, randomized controlled trials, non-randomized controlled trials, and mixed-method studies to answer the review question. As part of our search criteria, database searches were conducted from mHealth technology inception to July 2019. They were updated in March 2021 using PubMed, Science Direct, Google Scholar, MEDLINE, and CINAHL with full text via EBSCOhost databases. Reference lists of all included studies eligible for inclusion were also searched for relevant potential articles. Boolean terms (AND, OR) and MeSH (Medical Subject Headings) terms which formed part of the search strategy were used. The keywords used for the search included: “mHealth apps”, “mHealth devices”, “diagnostic”, “accuracy”, “sensitivity”, “specificity”, “health workers” and “sub-Saharan Africa” (Supplementary file S1). During the search, limitations such as date and language were removed. 

### 2.2. Study Selection

Following databases search for all the relevant studies, the principal investigator (EO) initially screened all titles of articles identified via the search strategy. All the eligible study titles were then exported to an Endnote X9 library specifically designed for this review. All duplicates identified were deleted, and the Endnote library was shared with the review team for abstract screening, which E.O. and P.N.V. performed in parallel. All discrepancies between the reviewers’ results following abstract screening were resolved through discussion until consensus was reached. Included studies following abstract screening were included for full-article screening performed by two reviewers, E.O. and P.N.V., independently. T.PM.-T., a third reviewer, was invited to resolve all the discrepancies in screeners’ results following the full-text screening. The screening was guided by the eligibility criteria presented below:

### 2.3. Eligibility Criteria

To ensure that all relevant evidence sources were identified and selected for our review, the study selection process was guided by the eligibility criteria specified under the inclusion and exclusion criteria. 

#### 2.3.1. Inclusion Criteria

The following criteria were used:

- Articles that presented evidence on Health Professionals using mHealth devices at POC diagnostics.
- Articles that presented evidence on diseases diagnosed at POC diagnostics.
- Studies that published evidence on other diagnostic tools linked to POC diagnostics.
- Articles published on the diagnostic accuracy of mobile-linked POC diagnostics.
- Articles that presented evidence from Sub-Saharan Africa.

#### 2.3.2. Exclusion Criteria

The following were excluded:

- Studies that presented evidence of patients using mHealth devices at POC diagnostics.
- Articles that reported evidence on typical diagnostic devices.
- Articles published on mHealth devices support treatment in appointment reminders, medication and treatment compliance, and others.
- Studies that showed evidence on mHealth for disease surveillance.
- Studies that published evidence on using mHealth for communication purposes.
- Articles that published evidence outside Sub-Saharan Africa.

### 2.4. Data Extraction

We designed a data extraction tool specifically for this review to extract all the relevant data from the included primary studies. The data for the analysis extracted from the included primary studies were organized in two sections: basic information and the primary study outcomes. The first section had the name of the author(s), date of publication, the aim of the study, country of research, study design, geographical settings, study setting, study population, sample size, type of mobile-linked POC diagnostics, key findings and conclusions. The second section also included true-positive values, false-positive values, true-negative values, false-negative values, sensitivity, specificity from each of the included primary studies, and a 2 × 2 table was constructed. E.O. and T.P.M.-T. independently conducted the included studies’ data extraction using the designed standard data extraction tool. A discussion resolved discrepancies between the reviewers’ responses until a consensus was reached.

### 2.5. Assessment of Methodological Quality

The Quality Assessment of Diagnostic Accuracy Studies 2 (QUADAS-2) tool was employed to assess the quality of all the included primary studies [34]. Quadas-2 is a well-structured tool recommended by the Cochrane Collaboration for determining diagnostic accuracy studies by evaluating them in four main domains: patient selection, index test, reference standard, and flow and timing [34]. The included primary studies’ risk of bias was comprehensively assessed independently by two reviewers (E.O. and T.P.M.-T.). All the disagreements in their assessment were resolved via a discussion. 

### 2.6. Data Analysis

The meta-analysis of diagnostic accuracy was considered for studies whose sensitivity and specificity had been evaluated. Statistical analyses were all performed using the R-based software Open Meta-Analyst [35]. A random-effects model (DerSimonian-Laird) was used to calculate the pooled sensitivity, specificity, and diagnostic odds ratio (DOR) with a 95% confidence interval (CI). A summary receiver operating characteristic curve (ROC) was constructed by plotting the individual and summary points of sensitivity and specificity to determine mobile devices’ overall diagnostic accuracy. Heterogeneity among the included primary studies was determined using *I*^2^ statistics where a score of 25% indicates low, a score of 50% represents moderate, and a score of 75% means high levels of heterogeneity [36]. A *p*-value < 0.05 was employed to demonstrate a statistically significant association in all the analyses.

## 3. Results 

### 3.1. Search

**Figure 1:**
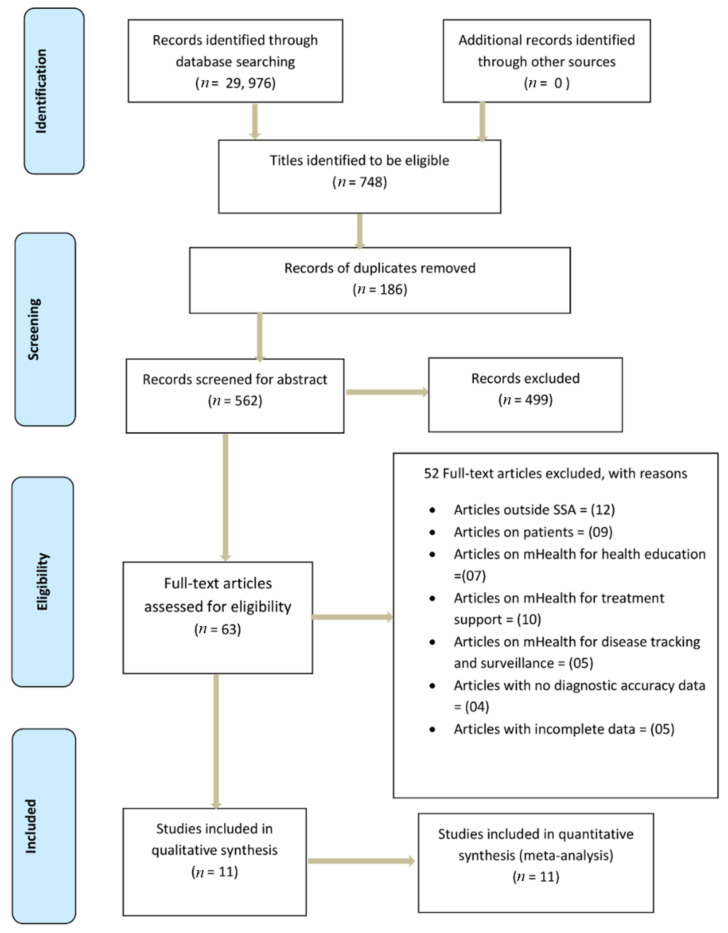
PRISMA flow chart showing literature search and selection of studies.

A total of 29,976 articles were identified from the combined search. Seven hundred forty-eight articles were eligible from the database search. One hundred eight-six duplicates were removed, leaving behind five hundred sixty-two articles suitable for abstract screening. A total of four hundred ninety-nine articles were excluded following the abstract screening. Sixty-three articles were eligible for full-text screening. Fifty-two of them were excluded, as illustrated in Figure 1, showing the PRISMA flow chart of literature search and selection of studies. Finally, 11 articles were included for data extraction and further underwent quantitative meta-analysis.

### 3.2. Characteristics of the Included Articles

Table 2 illustrates the characteristics of the included studies. A total of 11 articles were reviewed, and all underwent meta-analysis. Three of the included articles were conducted in Côte d’Ivoire [37,38], two in Ghana [39,40], two in Uganda [41,42], two in Sudan [43,44], one in Tanzania [45], and one in Ethiopia [46]. Sample sizes ranged from 50 to 1530 persons. Out of 11 studies, only 1 was a cohort study, and 10 were cross-sectional studies. All the included primary studies presented findings on the diagnostic accuracy of mobile-linked POC diagnostics in SSA. In terms of geographical settings, eight of the included studies were conducted in rural locations [37,38,39,40,41,45,46], while three were conducted in urban settings [42,43,44]. All the 11 included studies were conducted in English language from 2010 to 2017. 

### 3.3. Assessment of Risk and Applicability

**Figure 2:**
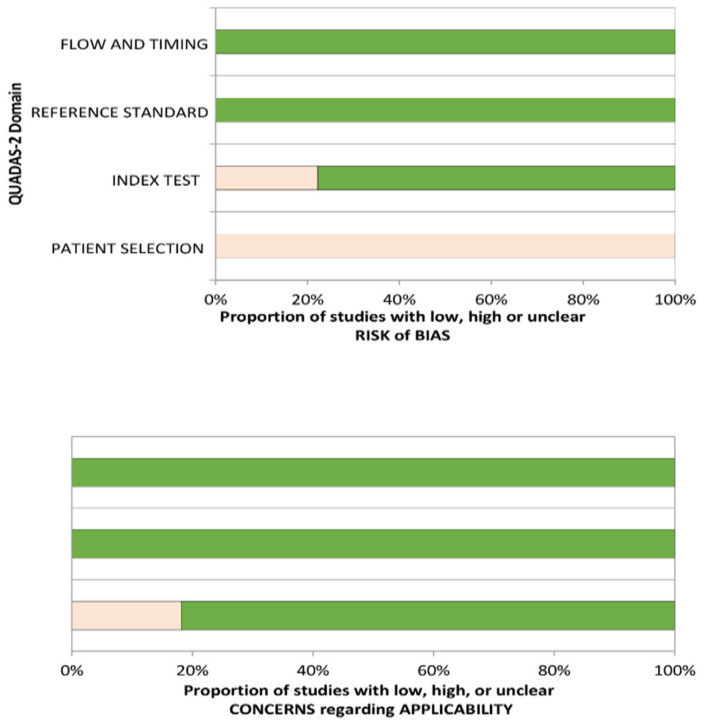
QUADAS-2 assessments of the included studies.

Table 3 shows the risk of bias and applicability concern assessment of the included studies using the QUADAS-2 tool. The results illustrate a range of findings in the included studies that employed QUADAS-2 as the quality assessment tool [34]. Participants’ enrolment in all the included studies was not based on random sampling or consecutive techniques regarding the patient selection domain but rather on a convenience approach. Even though it is highly possible that the convenience sampling technique could introduce a high-risk bias, it is unlikely to affect the diagnostic accuracy of mHealth devices. The reference standard domain was found to be at low risk of bias across all the included studies. The index test domain was at low risk of bias for most of the included studies. All the included studies were at low risk of bias in the flow and timing domain. However, all the studies included were at high risk of bias under the patient selection. Concerning the applicability assessment, nine of the included studies were at low risk of bias, while two were found to be a high risk of bias. Figure 2 displays the graphical results of the included studies from the QUADAS-2 assessment tool. 

### 3.4. Diagnostic Accuracy of Mobile-Linked Diagnostic Devices

**Figure 3:**
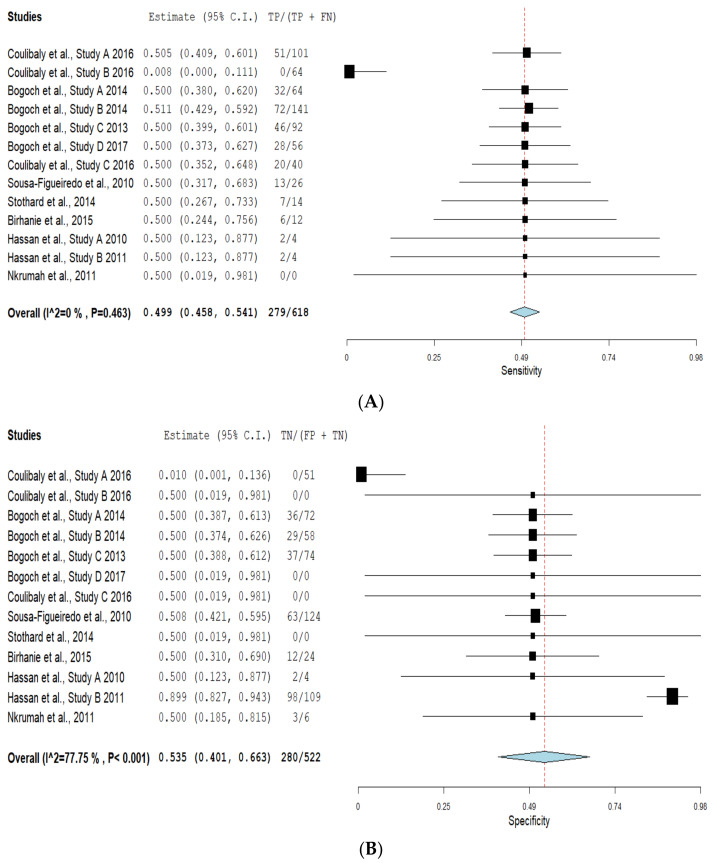
(**A**) Forest plots of pooled sensitivity and specificity estimates for all included studies of mobile-linked diagnostic devices; (**B**) Forest plots of pooled specificity estimates for all included studies of mobile-linked diagnostic devices.

**Figure 4:**
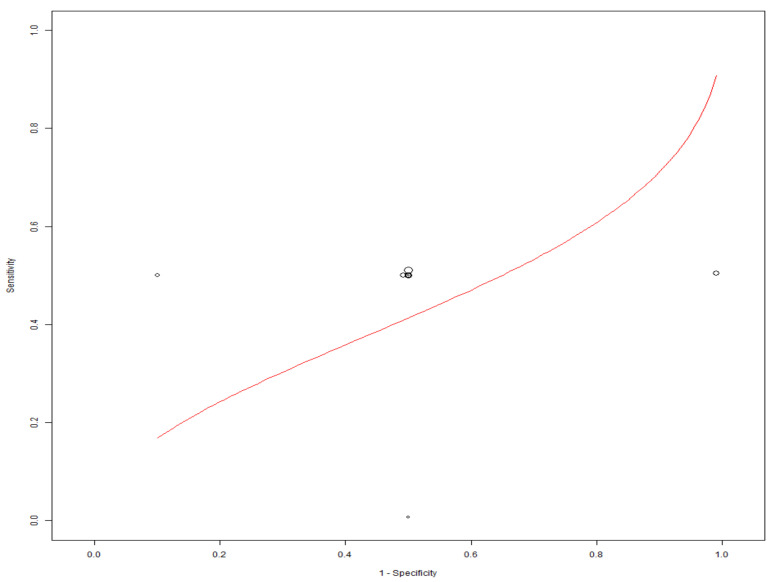
ROC graph of the included studies of mobile-linked POC diagnostic devices.

**Figure 5:**
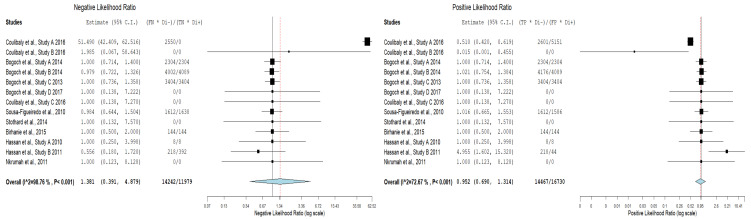
Negative likelihood ratio and positive likelihood ratio of the included studies of the mobile-linked POC diagnostic devices.

**Figure 6:**
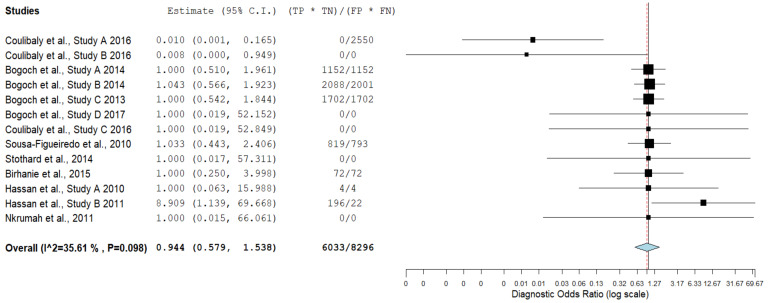
Diagnostic odds ratio forest plot of the included studies of mobile-linked diagnostic devices.

Table 4 illustrates true-positive, false-negative, false-positive, true-negative results and their corresponding sensitivity and specificity values for mobile-linked POC diagnostic devices for detecting disease conditions. The summary estimates of sensitivity and specificity of mobile-linked devices were 0.499 (95% CI: 0.458–0.541) and 0.535 (95% CI: 0.401–0.663), respectively (Figure 3A,B). The pooled estimates of specificity and sensitivity were statistically significant at the meta-analysis level. The individual pooled and summary estimates of sensitivity and specificity at the 95% CI region for all the included studies of mobile-linked POC diagnostic devices are presented in an ROC graph (Figure 4). The overall pooled estimates of the positive likelihood ratio (PLR) and negative likelihood ratio (NLR) were 0.952 (95%CI: 0.60–1.324) and 1.381 (95%CI: 0.391–4.879), respectively (Figure 5). Heterogeneity was determined as statistically insignificant, as I^2^ = 35.6% (*p* = 0.098) for the degree of inconsistency. The ROC curve analysis demonstrated a significantly moderate diagnostic performance of the mobile-linked POC diagnostic devices. The diagnostic odds ratio (DOR) for mobile-linked POC diagnostic devices’ accuracy was found to be OR = 0.944 (95% CI: 0.579–1.538) (Figure 6). Hence, the overall effect estimate of the study at the meta-analysis level was statistically insignificant.

## 4. Discussion

The evidence available from this study showed a moderate diagnostic accuracy of mobile-linked POC diagnostics in Sub-Saharan Africa. This systematic review’s objective was to evaluate the diagnostic accuracy of mobile-linked POC diagnostics in SSA. We found that mobile-linked POC diagnostics’ overall sensitivity for disease detections was 49.9%, and specificity was 53.5%. The meta-analysis results indicated a moderate diagnostic accuracy of mobile-linked POC diagnostic for disease detections in SSA. The ROC curve also confirmed the average diagnostic performance of these mobile-linked POC diagnostic devices. This means that mobile-linked POC diagnostics have less sensitivity and specificity abilities than the cut-off value of the gold standard described by the World Health Organization (WHO) [47]. We performed a sub-group analysis of the included studies to determine the rate of sensitivities and specificities of similar disease outcomes. A cursory examination of seven included studies that used mobile-linked POC diagnostic devices to detect malaria infections found moderate sensitivity and specificity estimates of 0.500 (95% CI: 0.352–0.648) and 0.500 (95% CI: 0.019–0.981) compared to the cut-off value of the gold standard light microscope described as an effective diagnostic tool [47]. 

The results also demonstrated that two studies that used mobile-linked POC diagnostic devices to detect *Schistosoma mansoni* found an average sensitivity estimate of 0.500 (95% CI: 0.380–0.620) and a low specificity estimate of 0.010 (95% CI: 0.001–0.136) compared to the gold standard conventional light microscope [47]. Again, the results illustrated that mobile-linked POC diagnostic devices for detecting *Schistosoma haematobium* infections found a low sensitivity estimate of 0.008 (95% CI: 0.409–0.601) and an average specificity estimate of 0.500 (95% CI: 0.019–0.981) compared to the gold standard conventional light microscope [47]. Additionally, the results indicated that two studies that used mobile-linked POC diagnostic devices to diagnose *Trichuris trichiura* infections found moderate sensitivity and specificity estimates of 0.511 (95% CI: 0.429–0.592) and 0.500 (95% CI: 0.388–0.612) compared to the gold standard light microscope [47]. These mobile-linked POC diagnostic devices providing moderate sensitivity and specificity estimates proved that such devices are below the cut-off point compared to the gold standard light microscope. The moderate diagnostic abilities of mobile-linked POC diagnostic devices for infectious and non-infectious diseases could also be attributed to the first-generation mobile phone microscopes employed in most of the included studies. 

A study conducted in some LMICs found the use of mobile phone fluorescence microscopy for detecting waterborne pathogens had an accuracy of 95%, which is not consistent with our study results [48]. Similar studies conducted in Finland and New Zealand illustrated that mobile phone microscopes exhibited high sensitivity for detecting soil-transmitted helminths and *Schistosoma*, which does not agree with our study results [49,50]. Luis Rosado et al. carried out another study in Portugal where s mobile phone microscope displayed higher sensitivity and specificity for diagnosing malaria infections, at variance with this study’s results [51]. A survey conducted in the USA by Paul Slusarewicz et al. revealed that mobile phone microscopes detected parasite eggs in mammalian feces with high sensitivity and specificity, which disagrees with this study’s findings [52]. A study conducted in Sweden revealed that mobile phone microscopes could be used extensively for clinical diagnostics when their sensitivities reach or exceed the 80% threshold [49]. Studies conducted in the USA have demonstrated that mobile handheld devices had a high diagnostic accuracy at POC diagnostics for detecting coronary stenosis and other disease conditions [26,53].

This review study included studies carried out in different geographical settings, given an exhaustive overview of the diagnostic accuracy of mobile-linked POC diagnostic devices in SSA. Date and language limitations were removed from this review study to capture all the essential literature on mobile-linked POC diagnostic devices’ diagnostic accuracy in SSA. Nonetheless, a piece of evidence on mobile-linked POC diagnostic devices’ diagnostic accuracy in SSA might have existed under different contexts that were not included in the study. This review was limited to studies that used quantitative methods, since this study focused on the diagnostic accuracy of mobile-linked POC diagnostic devices in SSA. The systematic review was also limited to studies conducted in SSA and could not be made to represent the entire world.

The results illustrated that most of the studies were conducted in rural settings where there is no access or little access to standard laboratory facilities. This will benefit such rural inhabitants by improving their health conditions if these activities are often conducted in such areas. The study results provided a moderate diagnostic yield of disease conditions and may not encourage healthcare professionals to rely on such devices to support healthcare provision continually. This means that more technologically advanced mobile-linked POC diagnostic devices, well validated with excellent sensitivities and specificities, should be made available to these healthcare professionals and other users. 

The results suggested that most of the studies that used first-generation mobile phones attached to microscopes provided a modest diagnostic yield of infectious and non-infectious diseases in resource-poor settings. We recommend future research on using low-cost technologically advanced mobile phone microscopes at POC in resource-constrained settings that may improve their diagnostic capabilities. The results also indicated that mobile-linked POC diagnostic devices’ diagnostic accuracy in detecting infectious and non-infectious diseases was found only in six SSA countries. We, therefore, encourage more countries in SSA to employ these mobile-linked POC diagnostic devices to assist in diagnosing more infectious and non-infectious diseases, especially in remote areas. 

The QUADAS-2 results showed a high risk of bias under the patient selection domain, which means that patients were selected not based on all consecutive or random sampling techniques. Employing any of these techniques means that eligible patients with suspected disease conditions were more likely to be chosen than those without any condition. In the included studies, inappropriate exclusions were made, which could have led to overoptimistic estimates of diagnostic accuracy. Studies that used consecutive patients with confirmed diagnoses were more likely to show greater sensitivity than those that included patients with suspected conditions. The low risk of bias under the index test domain for most of the included studies was because the index test results were interpreted without knowing the reference standard results. The low risk of bias under the reference standard domain means that the estimates of test accuracy were based on the reference standard with 100% sensitivity and specificity. It also means that the reference standard results were interpreted without the knowledge of the test index results. The low risk of bias in the flow and timing domain means that a reasonable time interval between index test and reference standard was given. This helped to determine the presence or absence of a target condition in the included studies. In cases where there is a bit of delay between the index test and reference standard, a possible misclassification of a disease condition may occur due to either recovery or deterioration of such condition.

## 5. Conclusions

Mobile-linked POC diagnostic devices can improve healthcare provision quality in clinical care to diagnose diseases in resource-constrained SSA areas. Current devices have been integrated slowly in routine clinical practice, with innovations such as mobile phone microscopes, machine learning, computer vision, and others that could assist in automatic diagnoses of diseases. The study results illustrated that mobile-linked POC diagnostic devices provided an average diagnostic yield in detecting infectious and non-infectious diseases in SSA. The study results further demonstrated that the first-generation mobile phones employed contributed to the moderate sensitivities and specificities in diagnosing infections in low-resourced SSA settings. Hence, we recommend that much more primary research should be carried out in SSA with mobile-linked POC diagnostic devices. These devices should be technologically advanced and well validated to provide sensitivities and specificities estimates to reach or exceed the 80% threshold. We also recommend that more mHealth diagnostics evaluation studies employ refined mHealth devices with excellent sensitivities and specificities to diagnose existing diseases in SSA.

## Figures and Tables

**Table 1:** PICO framework for determining the eligibility of the research question.

Determinants	Description
P-Population	Diseases such as communicable and non-communicable ones
I-Intervention	Type of mobile-linked POC diagnostics
C-Comparison	Other forms of diagnostic devices
O-Outcome	Diagnostic accuracy is defined as the actual results that contain both true positives (sensitivity) and true negatives (specificity) of a disease condition in a population [15].

**Table 2:** Characteristics of the included studies.

Author and Date	Country of Study	Aim of the Study	Geographical Setting (Urban/Semi-urban/Rural)	Study Setting	Study Design	Study Population (Diseases)	Type of mHealth Devices	Other Diagnostic Devices (Gold Standard)	Sample Size
Coulibaly et al., 2016a [41]	Côte d’Ivoire	To compare the accuracy of mobile phone and handheld devices to that of light microscopy to diagnose *Schistosoma haematobium*, *S. mansoni*, and intestinal protozoa infections in a community-based survey	Rural	Grand Moutcho community	Cross-sectional survey	*Schistosoma haematobium Schistosoma mansoni*, and Intestinal Protozoa Infections	Newton Nm1 reversed lens CellScope	Olympus Cx21 microscope	226
Bogoch et al., 2014 [42]	Côte d’Ivoire	To examine the utility of a novel commercial, portable light microscope and a simple mobile phone microscope to diagnose *S. mansoni*, *S. haematobium*, and soil-transmitted helminths.	Rural	Azaguié Makouguié	Cohort study	*Schistosoma mansoni*, *Schistosoma haematobium* and Soil-transmitted helminths	iPhone add-on, Newton Nm1	Olympus Cx21 microscope	180
Nkrumah et al., 2011 [43]	Ghana	To compare the novel Partec Rapid Malaria Test and the Binax Now Malaria Rapid Diagnostic Test with conventional Giemsa stain microscopy for malaria diagnosis in children at the clinical laboratory of a health facility in a rural endemic area of Ghana	Rural	Agogo Presbyterian hospital	Cross-sectional survey	Malaria (*Plasmodium falciparum)*	CyScope	Thick Giemsa Smear	263
Bogoch et al., 2017 [44]	Ghana	To test the performance of the handheld microscope in the diagnosis of *Schistosoma*.	Rural	Sorodofo–Abaasa Village	Cross-sectional survey	*Schistosoma haematobium*	Novel Mobile phone microscope	Olympus Cx21 microscope	60
Stothard et al., 2014 [45]	Uganda	To assess the diagnostic performance of the Newton Nm1 microscope towards malaria microscopy	Urban	Kampala	Cross-sectional study	Malaria (*Plasmodium* spp.)	Newton Nm1	Olympus Cx22 microscope	50
Sousa-Figueiredo et al., 2010 [46]	Uganda	To assess the diagnostic performance of the CyScope microscope and the lateral-flow Paracheck-Pf test as RDTs for malaria in children under five and in women	Rural	Bugoigo, Walukuba, Piida, Bugoto, Bukoba, Lwanika	Cross-sectional survey	Malaria (*Plasmodium* spp.)	CyScope	Thick Giemsa Smear	1530
Hassan et al., 2011 [47]	Sudan	To compare the performance of the CyScope fluorescence microscope with that of Giemsa-stained light microscopy for the diagnosis of malaria among pregnant women	Urban	Medani Maternity hospital	Cross-sectional study	Malaria (*Plasmodium falciparum)*	CyScope	Thick Giemsa Smear	128
Hassan et al., 2010 [48]	Sudan	To examine the specificity and sensitivity of the CyScope microscope compared to the gold standard of light microscopy	Urban	Sinnar hospital	Cross-sectional study	Malaria (*Plasmodium falciparum*)	CyScope	Thick Giemsa Smear	293
Bogoch et al., 2013 [49]	Tanzania	To compare the diagnostic accuracy of our mobile phone microscope with that of conventional light microscopy	Rural	Pemba Island	Cross-sectional survey	*Trichuris trichiura*	iPhone add-on	Olympus Cx21 microscope	199
Birhanie et al., 2015 [50]	Ethiopia	To assess the diagnostic performance of the Partec rapid malaria test regarding light microscopy for the diagnosis of malaria in Northwest Ethiopia	Rural	Gendewuha health center	Cross-sectional study	Malaria (*Plasmodium* spp.)	CyScope	Thick Giemsa Smear	180
Coulibaly et al., 2016b [51]	Côte d’Ivoire	To evaluate the “real-world” diagnostic operating characteristics of a handheld light microscope with mobile phone attachment integrated into a community-based screening program for malaria in rural Côte d’Ivoire	Rural	Grand Moutcho community	Cross-sectional survey	Malaria (*Plasmodium falciparum*)	Newton Nm1	Olympus Cx22 microscope	223

**Table 3:** Summary of methodological quality assessed with the QUADAS-2.

Risk of Bias					Applicability Concerns		
Author and Year of Publication	Patient Selection	Index Test	Reference Standard	Flow and Timing	Patient Selection	Index Test	Reference Standard
Bogoch et al., 2014	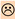	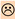	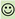	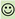	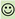	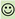	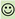
Coulibaly et al., 2016a	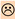	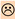	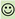	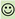	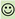	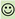	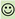
Coulibaly et al., 2016b	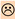	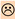	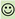	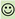	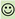	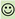	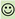
Bogoch et al., 2017	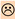	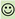	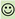	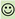	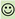	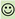	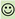
Stothard et al., 2014	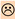	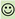	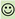	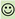	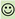	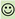	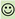
Bogoch et al., 2013	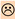	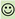	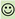	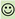	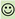	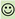	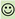
Sousa-Figueiredo et al., 2010	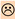	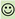	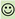	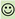	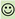	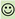	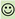
Birhanie et al., 2015	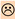	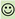	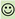	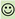	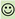	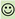	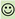
Hassan et al., 2010	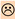	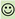	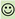	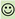	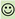	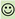	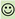
Hassan et al., 2011	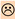	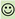	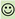	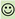	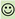	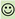	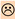
Nkrumah et al., 2011	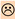	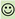	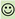	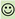	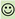	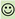	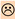

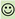
 Low Risk; 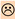 High Risk; ? Unclear Risk.

**Table 4:** Diagnostic accuracy of mobile-linked POC diagnostic devices.

Mobile Phone Microscope/CyScope									
Author, Date	Disease	Sensitivity (95% CI)	Specificity (95% CI)	PPV (95% CI)	NPV (95% CI)	TP (95% CI)	FP (95% CI)	TN (95% CI)	FN (95% CI)
Coulibaly et al., 2016a	*Schistosoma mansoni*	50.0 (25.4–74.6)	99.5 (97.0–100)	85.7 (42.0–99.2)	97.3 (93.9–98.9)	51.0	0.5	0.51	50
	*Schistosoma haematobium*	35.6 (25.9–46.4)	100 (96.6–100)	100 (86.7–100)	70.1 (63.1–76.3)	66.2	0.0	0.0	64.4
Bogoch et al., 2014a	*Schistosoma mansoni*	68.2 (60.1–75.5)	64.3 (35.1–87.2)	95.4 (89.5–98.5)	15.8 (7.5–27.9)	32.2	35.7	36.2	31.8
	*Trichuris trichiura*	30.8 (19.9–43.4)	71.0 (61.1–79.6)	40.8 (27.0–55.8)	61.2 (51.7–70.1)	71.5	29.0	29.0	69.2
Bogoch et al., 2013	*Trichuris trichiura*	*54.4(46.3–62.3)*	63.4 (46.9–77.4)	85.1 (76.4–91.2)	26.5 (18.4–36.6)	46.4	36.6	37.2	45.6
Bogoch et al., 2017	*Schistosoma haematobium*	72.1 (56.1–84.2	100.0 (75.9–100.0)	100.0 (86.3–100.0)	57.1 (37.4–75.0)	28.3	0.0	0.0	27.9
Coulibaly et al., 2016b	Malaria	80.2 (73.1–85.9)	100 (92.6–100.0),	100 (96.4–100.0)	65.6 (54.9–74.9)	20.0	0.0	0.0	19.8
Sousa-Figueiredo et al., 2010	Malaria	86.7 (79.3–92.2)	38.8 (33.6–44.1)	32.8 (27.7–38.3)	89.4 (83.4–93.8)	13.3	61.2	62.8	13.3
Stothard et al., 2014	Malaria	93.5 (78.6–99.2)	100 (82.4–100)	100 (88.1–100)	90.5 (69.6–98.8)	6.5	0.0	0.0	6.5
Birhanie et al., 2015	Malaria	93.8 (87.1–100)	87.9 (79.7–96.1)	86.4 (77.2–95.5)	94.6 (88.7–100)	6.3	12.1	12.2	6.2
Hassan et al., 2010	Malaria	98.2 (90.6–100)	98.3 (95.7–99.5)	93.3 (83.8–98.2)	99.6 (97.6–100)	1.8	1.7	1.72	1.8
Hassan et al., 2011	Malaria	97.6 (92.2–99.6)	89.1 (77.5–95.9)	94.1 (87.4–97.8)	95.3 (85.4–99.2)	2.43	10.9	98.2	2.4
Nkrumah et al., 2011	Malaria	100 (96.6–100)	97.4 (93.6–99.3)	96.4 (91–99)	100 (97.6–100)	0.0	2.6	2.63	0.0

## Data Availability

Not applicable.

## Supplementary Materials

The following are available online at https://www.mdpi.com/article/10.3390/diagnostics11061081/s1, Supplementary file S1: Results from the initial database search.

## Prospero Registration

CRD 42020155041.
