# Supplementary material for: Systematic Review and Meta-Analysis of the Diagnostic Accuracy of Mobile-Linked Point-of-Care Diagnostics in Sub-Saharan Africa
Source: Diagnostics (Basel). 2021 Jun 12;11(6):1081. doi: 10.3390/diagnostics11061081 (PMC8231511; doi:10.3390/diagnostics11061081)
Supplement: Supplementary file 1 [file diagnostics-11-01081-s001.zip › diagnostics-1254470-supplementary.pdf]

Additional file 1: Results from the initial database search

Dates of Search	Search Engine used	Keywords Search	Retrieved articles	Eligible articles
30 Jul 2019	PubMed	(((((((((("telemedicine"[MeSH Terms] OR "telemedicine"[All Fields] OR "mhealth"[All Fields]) AND ("technology"[MeSH Terms] OR "technology"[All Fields] OR "technologies"[All Fields])) OR (("telemedicine"[MeSH Terms] OR "telemedicine"[All Fields] OR "mhealth"[All Fields]) AND ("Appl Plant Sci"[Journal] OR "apps"[All Fields]))) OR (("telemedicine"[MeSH Terms] OR "telemedicine"[All Fields] OR "mhealth"[All Fields]) AND applications[All Fields])) OR ("telemedicine"[MeSH Terms] OR "telemedicine"[All Fields] OR ("mobile"[All Fields] AND "health"[All Fields]) OR "mobile health"[All Fields])) AND (("telemedicine"[MeSH Terms] OR "telemedicine"[All Fields] OR "mhealth"[All Fields]) AND ("instrumentation"[Subheading] OR "instrumentation"[All Fields] OR "devices"[All Fields] OR "equipment and supplies"[MeSH Terms] OR ("equipment"[All Fields] AND "supplies"[All Fields]) OR "equipment and supplies"[All Fields]))) AND (("point-of-care systems"[MeSH Terms] OR ("point-of-care"[All Fields] AND "systems"[All Fields]) OR "point-of-care systems"[All Fields] OR ("point"[All Fields] AND "care"[All Fields]) OR "point of care"[All Fields]) AND ("Diagnostics (Basel)"[Journal] OR "diagnostics"[All Fields]))) AND (("diagnosis"[MeSH Terms] OR "diagnosis"[All Fields] OR "diagnostic"[All Fields]) AND accuracy[All Fields])) AND ("sensitivity and specificity"[MeSH Terms] OR ("sensitivity"[All Fields] AND "specificity"[All Fields]) OR "sensitivity and specificity"[All Fields] OR "sensitivity"[All Fields])) AND ("sensitivity and	15,405	204

		specificity"[MeSH Terms] OR ("sensitivity"[All Fields] AND "specificity"[All Fields]) OR "sensitivity and specificity"[All Fields] OR "specificity"[All Fields])) OR (("health"[MeSH Terms] OR "health"[All Fields]) AND ("occupational groups"[MeSH Terms] OR ("occupational"[All Fields] AND "groups"[All Fields]) OR "occupational groups"[All Fields] OR "workers"[All Fields]))) AND ("africa south of the sahara"[MeSH Terms] OR ("africa"[All Fields] AND "south"[All Fields] AND "sahara"[All Fields]) OR "africa south of the sahara"[All Fields] OR ("sub"[All Fields] AND "saharan"[All Fields] AND "africa"[All Fields]) OR "sub-saharan africa"[All Fields]) AND ("1900/01/01"[PDAT] : "2019/07/30"[PDAT])		
30 Jul 2019	EBSCOhost (MEDLINE and CINAHL with full text)	mHealth technologies OR mHealth apps OR mHealth applications OR mobile health AND mHealth devices AND point of care diagnostics AND diagnostic accuracy AND sensitivity OR specificity AND health workers AND sub-Saharan Africa	1,646	110
31 Jul 2019	Google Scholar	mHealth technologies OR mHealth apps OR mHealth applications OR mobile health AND mHealth devices AND point of care diagnostics AND diagnostic accuracy AND sensitivity OR specificity AND health workers AND sub-Saharan Africa	427	86
31 Jul 2019	Science Direct	mHealth technologies AND mHealth devices AND point of care diagnostics AND diagnostic accuracy AND sensitivity OR specificity AND health workers AND sub-Saharan Africa	9,185	168
**Additional database search**				
08 Mar 2021	PubMed	mHealth technologies OR mHealth apps OR mHealth applications OR mobile health AND mHealth devices AND point of care diagnostics AND diagnostic accuracy AND sensitivity	261	42

		OR specificity AND health workers AND sub-Saharan Africa		
09 Mar 2021	Google Scholar	mHealth technologies OR mHealth apps OR mHealth applications OR mobile health AND mHealth devices AND point of care diagnostics AND diagnostic accuracy AND sensitivity OR specificity AND health workers AND sub-Saharan Africa	146	18
10 Mar 2021	EBSCOhost (MEDLINE and CINAHL with full text)	mHealth technologies OR mHealth apps OR mHealth applications OR mobile health AND mHealth devices AND point of care diagnostics AND diagnostic accuracy AND sensitivity OR specificity AND health workers AND sub-Saharan Africa	841	69
10 Mar 2021	Science Direct	mHealth technologies AND mHealth devices AND point of care diagnostics AND diagnostic accuracy AND sensitivity OR specificity AND health workers AND sub-Saharan Africa	2,065	51
